# TLR4-SIRT3 Mechanism Modulates Mitochondrial and Redox Homeostasis and Promotes EPCs Recruitment and Survival

**DOI:** 10.1155/2022/1282362

**Published:** 2022-07-04

**Authors:** Xiaotian Wang, Weidong Yao, Meihui Wang, Junhui Zhu, Liang Xia

**Affiliations:** ^1^Department of Clinical Medicine, School of Medicine, Zhejiang University City College, China; ^2^Biomedical Research (Therapy) Center, Sir Run Run Shaw Hospital, School of Medicine, Zhejiang University, China; ^3^Department of Cardiology, Sir Run Run Shaw Hospital, School of Medicine, Zhejiang University, China

## Abstract

The low survival rate of endothelial progenitor cells (EPCs) in vivo which are susceptible to adverse microenvironments including inflammation and oxidative stress has become one primary challenge of EPCs transplantation for regenerative therapy. Recent studies reported functional expression of toll-like receptor (TLR) 4 on EPCs and dose-dependent effects of lipopolysaccharide (LPS) on cellular oxidative stress and angiogenic properties. However, the involved mechanism has not yet been elucidated well, and the influence of TLR4 signaling on EPCs survival and function *in vivo* is unknown. In the present study, we observed the effects of LPS and TLR4/SIRT3 on EPCs mitochondrial permeability and intracellular mitochondrial superoxide. We employed the monocrotaline-induced pulmonary arteriolar injury model to observe the effects of TLR4/SIRT3 on the recruitment and survival of transplanted EPCs. We found the destructive effects of 10 *μ*g/mL LPS on mitochondrial homeostasis, and cellular viability was mediated by TLR4/SIRT3 signals at least partially, and the TLR4 mediates the early-stage recruitment of transplanted EPCs in pulmonary arteriolar inflammation injury; however, SIRT3 has more contribution to the survival of incorporated EPCs and ameliorated arteriolar remodeling in lung vascular tissue. The study provides insights for the critical role of TLR4/SIRT3 in LPS-induced oxidative stress and mitochondrial disorder in EPCs *in vitro* and *in vivo*. The TLR4/SIRT3 signaling is important for EPCs resistance against inflammation and oxidative stress and may represent a new manipulating target for developing efficient cell therapy strategy.

## 1. Introduction

By contributing to reendothelialization and neovascularization, endothelial progenitor cells (EPCs) play a major role in maintaining the normal structure and function of vessels. In past decades, it has become one of the highlights of researches to promote the regenerative capacity of these precursor cells for autologous transplantation for cardiovascular disease [1, 2]. However, due to the unfriendly microenvironments such as hypoxia and inflammation, the transplanted EPCs suffer from low survival rate in vivo and produce limited therapeutic effects.

As a pattern recognition receptor and type I transmembrane protein, toll-like receptors (TLRs) are mainly expressed in immune cells such as monocytes and dendritic cells and identify the surface of pathogenic microorganisms, transfer the signals to the cells, and participate in the innate and adaptive immunity of the body [3]. Among at least 11 different subtypes of TLRs, TLR4 recognizes various molecules including lipopolysaccharide (LPS), heat-shock proteins (HSP), hyaluronic acid, fibrinogen, *β*-defensin 2, and the fusion protein from respiratory syncytial virus [4–6].

Previous studies showed that TLR4 signaling involved in a variety of inflammatory and hypoxic conditions, including atherosclerosis, reperfusion injury, cardiac hypertrophy, pulmonary hypertension, and sepsis [7–11]. Recently, functional TLR4 was also found expressed on human EPCs [12–14], which can induce the downstream signals transduction and modulate the EPCs functional performance, including migration, adhesion, and angiogenesis [13, 14]. Moreover, in our previous study, we found that the pretreatment with an appropriate dose of LPS (1 *μ*g/mL) can trigger the NF-*κ*B p65 signaling pathway and prevent the ROS generation and apoptosis in human EPCs in vitro by a TLR4-dependent way [15].

However, whether the mitochondrial mechanism, which usually crucial for cellular redox homeostasis, mediates the effects of TLR4 signaling on EPCs has never been shown before. Furthermore, the influence of TLR4 signaling on EPCs survival and function in vivo with the challenge of inflammatory and oxidative microenvironment is not recognized yet.

## 2. Methods and Materials

### 2.1. Cell Isolation and Culture

The blood samples were obtained from healthy volunteers and were treated and tested individually in independent experiments. The peripheral blood mononuclear cells (PBMNCs) were isolated by density-gradient centrifugation with Ficoll separating solution (Cedarlane Laboratories Ltd., Canada). PBMNCs were plated into fibronectin-coated six-well plates at a density of 2×10^5^/cm^2^ with 1.5 mL EGM-2MV medium (Lonza Group, Switzerland) for each well, supplemented with 10% fetal bovine serum, vascular endothelial growth factor, fibroblast growth factor-2, epidermal growth factor, insulin-like growth factor, and ascorbic acid. Adherent MNCs cultured for 7-14 days in the above conditions were used to derive EPCs.

For fluorescent staining, adherent cells were first incubated with 10 mg/mL 1,1-dioctadecyl-3,3,3,3-tetramethylindocarbocyanine-labeled acetylated low-density lipoprotein (DiI-Ac-LDL) (Molecular Probes, US) at 37°C for 2 h, then fixed with 2% paraformaldehyde for 10 min, followed by incubation with 10 *μ*g/mL fluorescein isothiocyanate (FITC)-labeled Ulex europaeus agglutinin lectin (UEA)-1 (Sigma-Aldrich, US) at 37°C for 1 h. The cells were identified under a fluorescence microscope. Double-positive fluorescence was identified as differentiating EPCs.

To investigate the expression of TLR4, EPCs after 7 days culture on six-well plates were preconditioned with various concentrations of LPS (0, 1 or 10 *μ*g/mL; Sigma-Aldrich) for 12 h. Then single cell suspension of 10^6^ EPCs/mL in ice cold PBS with 10% FCS and 0.1% sodium azide from each group were prepared and incubated with mouse monoclonal antibody (R&D System, US) against human TLR4 for 40 min on ice, followed by washes with PBS and incubation with FITC-labeled rabbit antimouse IgG (Abcam, UK) for 30 min at 4°C in the dark. Cells were then washed with PBS, fixed in 1% paraformaldehyde, and analyzed on the flow cytometer (FACSCalibur, Becton Dickinson, US). Each analysis included 10000 events. Isotype-matched primary antibody (R&D System, US) was used as controls.

### 2.2. Intracellular Reactive Oxygen Species Assay

Generation of ROS in EPCs was measured by the membrane permeable indicator 2′,7′-dichlorodihydrofluorescein diacetate (H_2_DCFDA) (Invitrogen; Thermo Fisher Scientific, Inc., US). The EPCs were cultured in 6-well plates and either treated with various concentrations of LPS (1 or 10 *μ*g/mL) for 12 h or pretreated with 1 *μ*M TAK-242 (MedChem Express, US) for 1 h with subsequent exposure to different concentrations of LPS, followed by serum-free starvation for 24 h. For measurement of intracellular ROS, the EPCs were incubated with 10 *μ*M H_2_DCFDA in serum-free EGM-2MV medium at 37°C for 30 min and then washed twice with PBS. After trypsinized and resuspended in serum-free medium, the cells were analyzed with flow cytometry (FACSCalibur, Becton Dickinson, US) at the wavelengths of 488 and 525 nm for excitation and emission, respectively. The production of ROS was determined in terms of mean fluorescence intensity (MFI). For each group, 5 replicates were performed.

### 2.3. Assessment of Intracellular Mitochondrial Superoxide

The mitochondrial superoxide was detected by the MitoSOX™ Red indicator (Invitrogen, US). According to the manufacturer's recommended protocol and reference, the MitoSOX™ reagent at the working concentration was added into the six-well plates to cover the EPCs. After 10-minute incubation at 37°C in the dark, the EPCs were washed for three times by Hanks buffer. Images were obtained by a fluorescence microscope using a green excitation light. For each group, 5 replicates were performed. Five predetermined fields of view of each well were observed, and representative images were shown.

### 2.4. Assessment of Mitochondrial Permeability Transition Pore (mPTP) Activity

The mPTP Assay Kit (Genmed, US) was used to evaluate the mPTP activity, wherein fluorescence quenching by cobalt ions increased when the mPTP activity increased. The staining and fixation of cells were performed according to the manufacturer's protocol. Briefly, the culture medium was removed, and cells were washed by PBS for 2 times. Then, 1 mL calcein AM staining solution with 10 *μ*L CoCl_2_ fluorescence quenching solution were added to one well of 6-well plate and incubated at 37°C for 30 min in darkness and then replaced by fresh culture medium preheated to 37°C and incubated at 37°C for 30 min in darkness again. After washing the cells with PBS for 2-3 times, the detection buffer was added, and then cells were observed by a fluorescence microscope (Olympus IX71, JP). Five replicates were performed for each group.

### 2.5. Western Blot Analysis

EPCs were lysed in Western and IP cell lysate (PH0406, Phygene, China) on ice for 15 min. The total protein was collected after centrifugation. Protein concentration was measured by BCA assay kit. Equal quantification of protein (20 *μ*g) was applied in the 15% SDS-polyacrylamide gel and then transferred to polyvinylidene fluoride (PVDF) membranes. The membranes were blocked by 5% milk for 1 hour at room temperature and then incubated with the primary antibody against SIRT3 (rabbit against human, ab217319) or GAPDH (rabbit against human, ab181603, Abcam, UK) overnight at 4°C. After being washed three times in TBS buffer, the membrane was incubated with horseradish peroxidase (HRP)-conjugated secondary antibodies at room temperature for 2 hours. The proteins were visualized with enhanced chemiluminescence (Amersham, Israel) on a LAS-4000 image reader system (Fujifilm, JP).

### 2.6. SIRT3 Deacetylase Activity Assay in Human EPCs

The deacetylase activity of SIRT3 was determined using a SIRT3 Fluorescent Activity Assay/Drug Discovery Kit (Enzo Life Sciences, US) according to the manufacturer's instruction. Briefly, 5 *μ*g mitochondrial extract obtained by Cell Mitochondria Isolation Kits (C3601, Beyotime, China) was incubated with the FLUOR DE LYS®-SIRT2 buffer at 37°C for 60 min, followed by incubation with FLUOR DE LYS® Developer II buffer at 37°C for 40 min. The fluorescence intensity was measured with a microtiter-plate fluorimeter, with 360-nm excitation wavelength and 460-nm emission wavelength. The “blank” value of assay buffer was subtracted from all other values.

### 2.7. Measurement of NADPH Oxidase Activity

To measure NADPH oxidase activity in EPCs, the lucigenin-derived enhanced chemiluminescence assay (Beyotime, China) was used. After 24 h serum-free starvation, EPCs were incubated with LPS (1 or 10 *μ*g/mL) and then washed two times with ice cold PBS (pH 7.4) and centrifuged at 2,000 g for 5 min at 4°C. The EPCs were resuspended in ice cold PBS containing 1 mmol/L ethylene glycol tetraacetic acid, protease inhibitors, and 150 mmol/L sucrose and lysed. The total protein concentration was adjusted to 1 mg/mL with the employment of Bradford assay. For each sample, 100 *μ*L protein including 2.5 *μ*mol/L lucigenin was measured over 6 min in quadruplicate with NADPH (100 *μ*mol/L) as a substrate in a luminometer counter (Centro LB 960; Berthold Technologies GmbH & Co. KG, Germany).

### 2.8. Cell Viability and Proliferation Assays

Cell viability assessment was performed with a Cell Counting Kit-8 (CCK-8; CK04, Dojindo, JP). After treatment with PBS or LPS (1 or 10 *μ*g/mL), 10 *μ*L CCK-8 solution was added into each well, and the EPCs were further cultured for 4 h at 37°C. Absorbance at 450 nm was measured by a spectrophotometer (BioTek, US).

Cell proliferation was measured by EdU incorporation assay. After treatment with LPS for 20 hours, EPCs were treated with 10 *μ*M of EdU (Invitrogen) for another 4 hours. The cells were then fixed with 4% paraformaldehyde, and EdU labeling was detected using Click-iT EdU Imaging Kits (Invitrogen) per the manufacturer's instructions. DAPI was used for nuclear staining. Images from five predetermined fields per well were taken, and cell proliferation was determined by calculating the ratio of EdU-positive cells to total DAPI-positive cells.

### 2.9. Lentivirus Production and Transfection

The SIRT3 overexpression was completed using recombinant lentivirus vectors containing the overexpression plasmid of the corresponding gene (GeneChem, China). The EPCs were collected after 7 days culture and reseeded in 6 well plates at a density of 5 × 10^5^/mL. The media volume for each well was 1 mL. After 24-48 hours of culture, when the cell confluence arrived 30-40%, 100*μ*L of 1 × 10^8^ TU/mL virus diluent was added into the media, and polybrene was used with a final concentration of 5*μ*g/mL. The media was changed after 8-12 hours, and the culture continued for 72 hours, followed by a western blot assay for efficiency determination.

### 2.10. Oligonucleotide Transfection

RNA interference was conducted using the Oligofectamine reagent (Invitrogen). And cultured EPCs were transfected with the 100 nM SIRT3 siRNA or negative control siRNA (GenePharma, Shanghai, China) according to the instruction. Cells had 60%-70% confluence on the day of transfection. After transfecting for 48hours, the knockdown efficiency was tested by western blot.

### 2.11. Animal Models of Pulmonary Arteriolar Injury

Male immunodeficient (F344/N rnu/rnu) nude rats of 12 weeks old (weighing 163 to 216 g) were randomly assigned into 4 groups: control group received a subcutaneous saline injection on day 1 and 1 mL saline through right jugular vein on day 21 (*n* = 4); monocrotaline (MCT) group received a subcutaneous injection of 60 mg/kg MCT on day 1 and 1 mL saline through right jugular vein on day 21 (*n* = 4); MCT/EPCs group received 60 mg/kg MCT on day 1 and human EPCs (1 × 10^6^ in 1 mL saline) through right jugular vein on day 21 (*n* = 4); and MCT/(EPCs+LetSIRT3) group received 60 mg/kg MCT on day 1 and 1 × 10^6^ LetSIRT3 transfected human EPCs on day 21 (*n* = 4). On day 42 after the EPC injection, respectively, the animals were killed. The right lungs were fixed by 10% formaldehyde and then paraffin embedded and sectioned for histological analysis. All the experiments were performed following the Helsinki Guideline and the Guidelines for Animal Experiments of Zhejiang University after approval from the Ethics Committee for Animal Experiments at Zhejiang University (No. ZJU201523505) and Medical Ethics Committee of the Sir Run Run Shaw Hospital of Zhejiang University (No. 20150227-20).

### 2.12. EPC Labeling and Fluorescence Assay

For this experiment, rats were divided into 5 groups: control/EPC group received a subcutaneous saline injection on day 1 and human EPCs (1 × 10^6^ in 1 mL saline) through right jugular vein on day 21 (*n* = 8); monocrotaline (MCT)/EPC group received a subcutaneous injection of 60 mg/kg MCT on day 1 and human EPCs (1 × 10^6^ in 1 mL saline) through right jugular vein on day 21 (*n* = 8); MCT/(EPC + TAK-242) group received MCT on day 1 and TAK-242 pretreated human EPCs on day 21 (*n* = 8); MCT/(EPC+ LetSIRT3) group received MCT on day 1 and LetSIRT3 transfected EPCs on day 21 (*n* = 8); and MCT/(EPC+ siSIRT3) group received MCT on day 1 and siSIRT3 transfected EPCs on day 21 (*n* = 8). Before transplantation, EPCs for each group were incubated for 40 minutes with 10 *μ*mol/L vital cytoplasmic fluorescent dye, CMTMR (5-(and-6)-(((4-chloromethyl)benzoyl)amino) tetramethylrhodamine, Molecular Probes, US), and then 1 × 10^6^ labeled cells were injected into the pulmonary circulation of rats on day 21 after saline or MCT injection. The right lungs were collected and sampled for fluorescent assay 2 or 9 days later. Fluorescence analysis was performed on frozen sections from the middle region of right lungs. The EPCs were detected by the red fluorescence of CMTMR. Tissue sections were examined by using laser confocal microscopy (LSM 510, Zeiss, Germany).

### 2.13. Histologic Analysis of Pulmonary Arterioles

Three *μ*m lung sections were stained by Masson's Trichrome Stain, by which vascular smooth muscle was indicated pink, fibril tissues green, and cellular nucleus stain brown. Then the vessels in the lung tissues were observed under a light microscope (×200 magnification). We analyzed the medial wall thickness of the pulmonary arterioles in the middle region of the right lungs (20 intra-acinar arterioles/rat, four rats for each group). The medial wall thickness was expressed as follows: %wall thickness = [(medial thickness in long axis + medial thickness in short axis)/(external diameter in long axis + external diameter in short axis)] × 100. Representative sections were photographed. All evaluation was performed by a blind investigator under light microscope.

### 2.14. Statistical Analysis

Data are expressed as mean ± SD from at least three independent experiments. Statistical analysis was performed by the two-tailed *t*-test or ANOVA for multiple comparisons. *P* < 0.05 was considered as statistical significance. SPSS 13.0 software (IBM, Chicago, IL, USA) was used for statistical analysis.

## 3. Results

### 3.1. Identification of Human EPCs and TLR4 Expression

**Figure 1:**
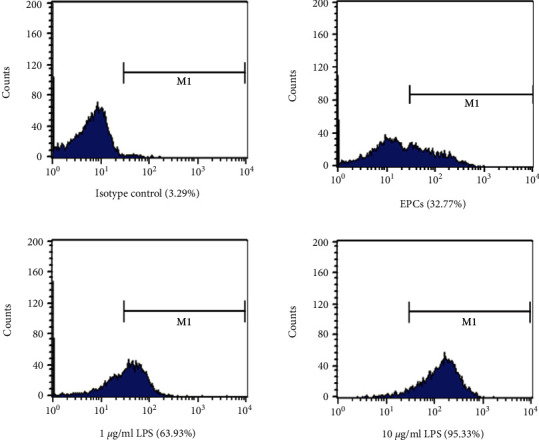
Identification of TLR4 expression in human EPCs. TLR4 expression on EPCs was detected by FACS. EPCs after 7 days culture were induced by various concentrations of LPS (1 or 10 *μ*g/mL) for 12 h. Then the EPCs were harvested and incubated with mouse anti-TLR4 antibody, followed by FITC-labeled rabbit antimouse IgG. FACS showed that the expression rate of TLR4 in human EPCs can be upregulated by LPS from 32.77% to 63.93% (1 *μ*g/mL) or 95.33% (10 *μ*g/mL).

EPCs were isolated from human peripheral blood by density gradient centrifugation. As viewed under the inverted microscopy, adherent cells were round shaped on day 2. After 4 days culture, they exhibited the typical spindle-like shape. After 7-14 days of continuous culture, the cells developed cobblestone and vortex-like appearance and displayed capability to take up Dil-Ac-LDL and bind to FITC-UEA-1 (Supplementary 1). As demonstrated in Figure 1, flow cytometry analysis showed that the expression rate of TLR4 in human peripheral EPCs was 32.77%, which can be further induce to 63.93% by 1 *μ*g/mL LPS or 95.33% by 10 *μ*g/mL LPS.

### 3.2. LPS Induces Mitochondrial Dysfunction and Superoxide Generation through TLR4

**Figure 2:**
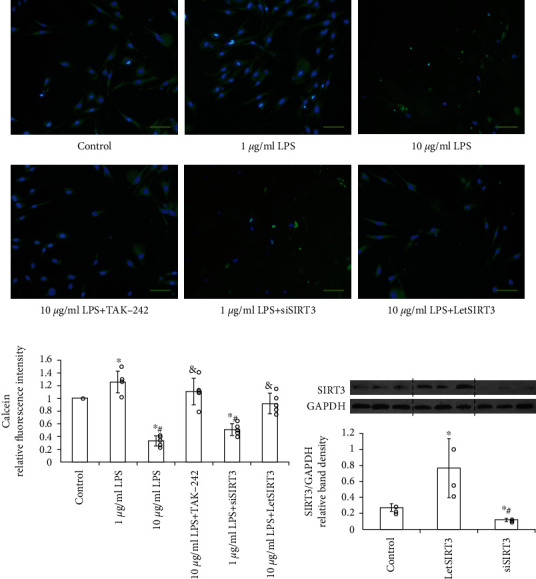
LPS induces mitochondrial permeability through TLR4. High-dose LPS (10 *μ*g/mL) treatment induced mitochondrial dysfunction via prolonged mPTP activation in EPCs, which can be ameliorated by TLR4-specific blocker TAK-242 or SIRT3 overexpression (LetSIRT3). (a) Representative fluorescent images with calcein staining (green) and (b) quantitative analysis of fluorescence intensity are shown here (*n* = 5 per group, results were expressed as mean ± SD). Cell nuclei are stained by DAPI. (Scale bars 100 *μ*m). ∗*p* < 0.05 vs. control; #*p* < 0.05 vs. 1 *μ*g/mL LPS; &*p* < 0.05 vs. 10 *μ*g/mL LPS. (c) The western blot assay demonstrated the efficiency of siRNA-induced knock down or lentiviral transfection-induced overexpression of SIRT3 in EPCs (*n* = 3). ∗*p* < 0.05 vs. control; #*p* < 0.05 vs. LetSIRT3 group.

In a previous study, we have found that pretreatment of EPCs with an appropriate dose of LPS (1 *μ*g/mL for 12 h) can markedly attenuate the cellular ROS production and thereafter reduced the apoptosis. However, the effects of LPS on mitochondria, as the most important source of intracellular oxygen free radical and proapoptotic molecules [16], have never been reported. Here, we firstly observed the change of mitochondrial membrane permeability reflected by the mitochondrial permeability transition pore (mPTP) dynamics. The fluorescence quenching of cobalt ions accelerates with the increase of mPTP activation. As compared with PBS or 1 *μ*g/mL LPS, 10 *μ*g/mL LPS pretreatment significantly reduced the retaining of Calcein (Figures 2), which indicates the prolonged mPTP activation. To investigate the role of TLR4 in LPS-induced mPTP change, TLR4 was blocked by specific antagonist TAK-242. This reversed the alteration induced by 10 *μ*g/mL LPS (Figures 2), indicating that TLR4 may mediate the LPS-induced mitochondrial dysfunction.

**Figure 3:**
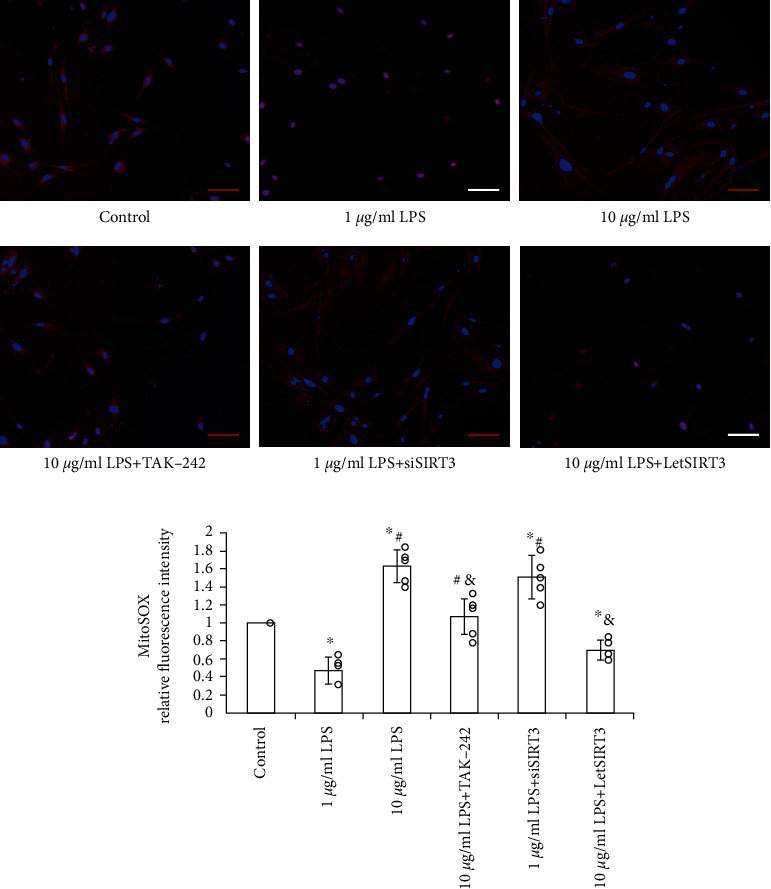
LPS induces mitochondrial superoxide generation through TLR4. MitoSOX-labeled (red) mitochondrial superoxide level was measured and calculated in EPCs (*n* = 5 per group, results were expressed as mean ± SD). While low dose LPS (1 *μ*g/mL) precondition reduced superoxide level in EPCs, the high dose (10 *μ*g/mL) induced mitochondrial superoxide accumulation significantly, which can be alleviated to a great extent by TLR4-specific antagonist TAK-242 or reversed by SIRT3 overexpression (LetSIRT3). (a) Representative fluorescent images and (b) quantitative analysis of fluorescence intensity are shown here (*n* = 5, mean ± SD). Cell nuclei are stained by DAPI. (Scale bars 100 *μ*m). ∗*p* < 0.05 vs. control; #*p* < 0.05 vs. 1 *μ*g/mL LPS; &p < 0.05 vs. 10 *μ*g/mL LPS.

Then the MitoSOX was utilized as the detector for labeling mitochondrial superoxide generation. After 1 *μ*g/mL LPS precondition for 12 hours, mitochondrial superoxide was sharply reduced. By contrast, incubation with 10 *μ*g/mL LPS promoted the superoxide generation considerably. Furthermore, this alteration was ameliorated in TLR4-blocked EPCs (Figures 3), which implies the TLR4 involvement in the LPS-induced mitochondrial oxidative stress.

### 3.3. LPS Induces the Changes in SIRT3 Expression and Activity through TLR4

**Figure 4:**
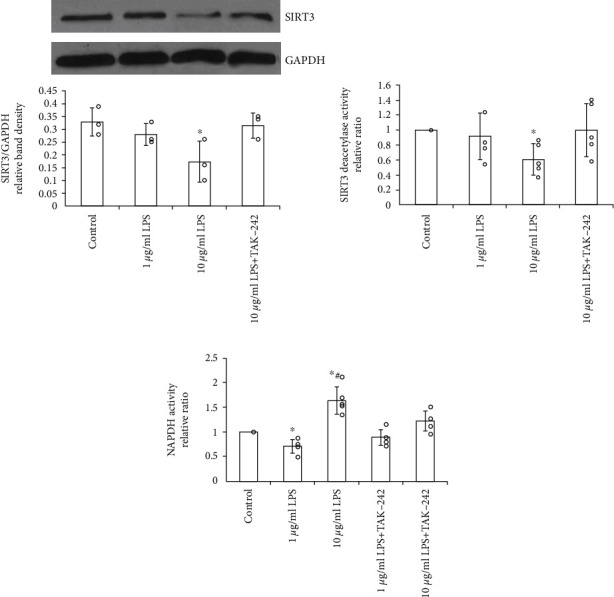
LPS induces the changes in SIRT3 expression and activity through TLR4. Decreased protein and deacetylase activity levels by high-dose LPS (10 *μ*g/mL) can be abrogated significantly by TLR4 blocking in EPCs. (a) Representative western blotting assay and quantitation of the level of SIRT3 protein. Data are presented as mean ± SD (*n* = 3) (b). The analysis of SIRT3 deacetylase activity by using mitochondrial fractions (*n* = 5). (c) Pretreatment with LPS (10 *μ*g/mL) significantly elevated the NADPH oxidative activity in a TLR4-dependent way (*n* = 5). ∗*p* < 0.05 vs. control; #*p* < 0.05 vs. 1 *μ*g/mL LPS.

Intact SIRT3 function is very critical for maintaining mitochondrial redox homeostasis and physiological function. Defect or dysfunction of SIRT3 has been found involved in various oxidative stress-related diseases [17–19]. We treated human EPCs with LPS for 12 hours *in vitro* and detected SIRT3 protein level and deacetylase activity. Western blot results showed a dose-dependent influence on SIRT3 protein levels induced by LPS (Figure 4(a)). Similarly, SIRT3 deacetylase activity was correlated with the protein levels after LPS treatment (Figure 4(b)). We also observed that when TLR4 in EPCs was blocked, the expression and deacetylase activity of SIRT3 restored after LPS treatment.

To our knowledge, the NADPH oxidase, as a crucial member of the mitochondrial oxidation-reduction network, is one of the downstream molecular targets of SIRT3 [19, 20]. In the measurement of NADPH oxidase activity by chemiluminescence, pretreatment of 1 *μ*g/mL LPS significantly decreased NADPH oxidative activity which can be raised sharply by 10 *μ*g/mL LPS (Figure 4(c)). Furthermore, the LPS-induced alterations of NOX activities can be alleviated by TAK-242 dramatically, implying the contribution of TLR4 to SIRT3 regulation under LPS stimulation.

### 3.4. SIRT3 Mediates the Change of Mitochondrial Function and Cellular Oxidative Stress under LPS Activation and Regulate EPC Survival and Proliferation

**Figure 5:**
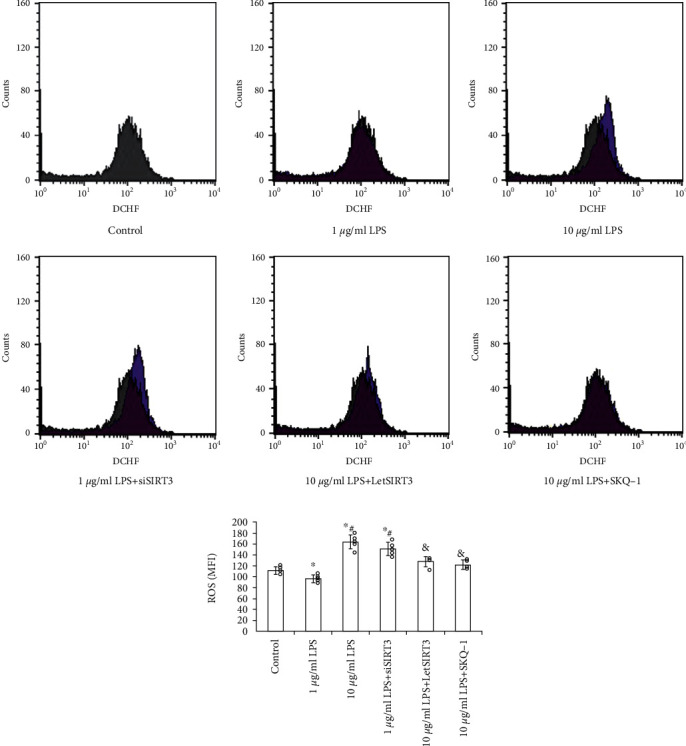
The provoking effect of 10 *μ*g/mL LPS on ROS generation in EPCs can be reversed by SIRT3 upregulation or mitochondrial antioxidant SKQ1. Meanwhile the protective effect of 1 *μ*g/mL LPS can be abolished by SIRT3 silence. (a) Flow cytometric analysis of cellular ROS by using DCF fluorescence. The graph of control has been overlapped with each other groups. (b) Histogram showing MFI of each group. Data are presented as mean ± SD (*n* = 5). ∗*p* < 0.05 vs. control; #*p* < 0.05 vs. 1 *μ*g/mL LPS; &*p* < 0.05 vs. 10 *μ*g/mL LPS.

To investigate whether SIRT3 could antagonize LPS-induced ROS accumulation, mitochondrial dysfunction, and cellular capacity injury in EPCs, SIRT3 in EPCs was significantly knocked down by targeted siRNA (siSIRT3) or overexpressed by recombined lentivirus of SIRT3 (LetSIRT3) transfection, which significantly suppressed or increased the expression of SIRT3 without mutual interference. Compared with the untreated group, the addition of LPS caused ROS accumulation (Figure 5(a)), mitochondrial dysfunction (Figure 2), and superoxide generation (Figure 3) in EPCs significantly at 10 *μ*g/mL. The similar effects can be obtained by SIRT3 knockdown in EPC. However, SIRT3 overexpression groups displayed alleviated oxidative stress (Figure 5), which mimicked the effect of mitochondrial antioxidant SKQ1. And SIRT3 overexpression can markedly abrogate the high-dose LPS-induced rise of mitochondrial permeability and superoxide generation (Figures 2 and 3).

**Figure 6:**
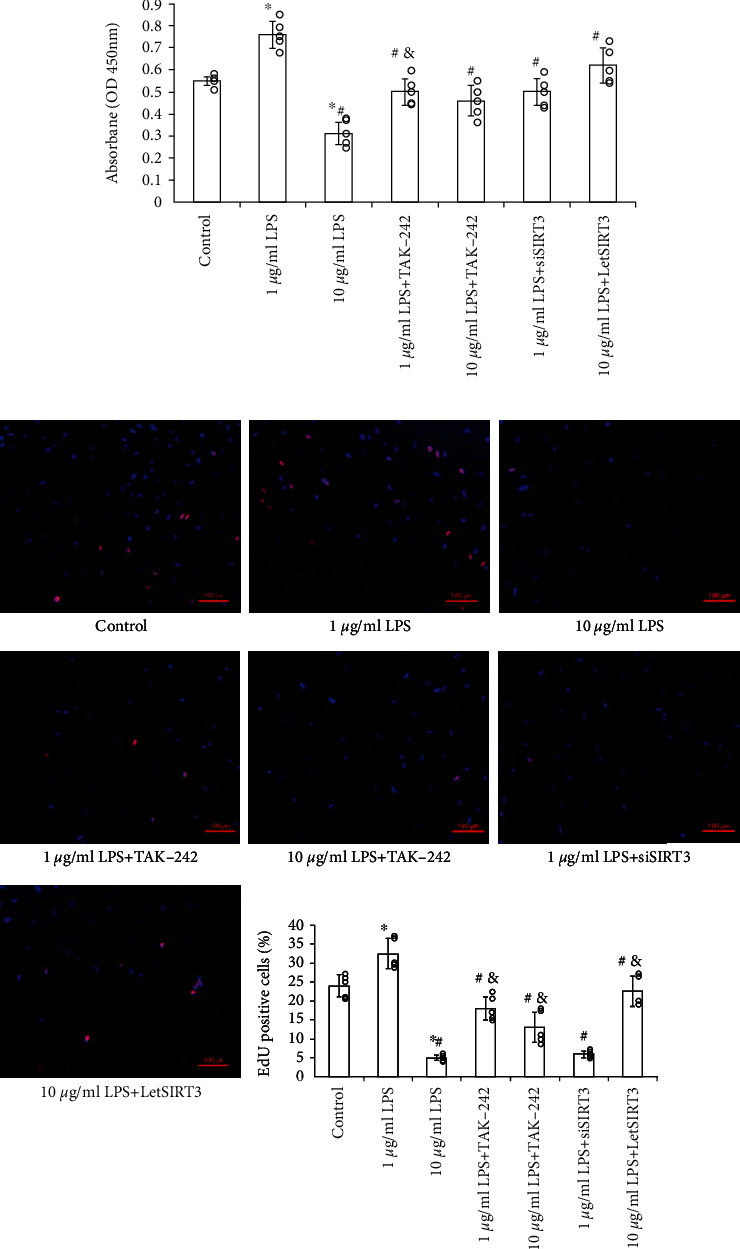
The effects of LPS on EPCs viability and proliferation were mediated by TLR4-SIRT3 mechanism. (a) Cell viability was examined by the absorbance of CCK-8. (b) Cell proliferation was detected by EdU staining, and the nuclei was stained by DAPI. The double positive cells were quantitated under fluorescence microscope (Scale bars 100 *μ*m). Data are presented as mean ± SD (*n* = 5). ∗*p* < 0.05 vs. control; #*p* < 0.05 vs. 1 *μ*g/mL LPS; &*p* < 0.05 vs. 10 *μ*g/mL LPS.

Additionally, though 10 *μ*g/mL LPS led to significantly suppress survive and proliferation (Figure 6), both the TAK-242 and SIRT3 overexpression showed the antagonistic effect. Especially the SIRT3 overexpression almost reversed such impact. Moreover, the suppression of SIRT3 contributed to the enhanced oxidative stress and cellular dysfunction. These results suggest that SIRT3, at least partly, mediated the LPS-TLR4-induced ROS accumulation, mitochondrial dysfunction and contributed to cellular capacity injury.

### 3.5. Upregulation of SIRT3 Increases the Survival of EPCs in Lung Tissue after MCT-Induced Injury and Ameliorates the Pulmonary Arteriolar Remodeling

**Figure 7:**
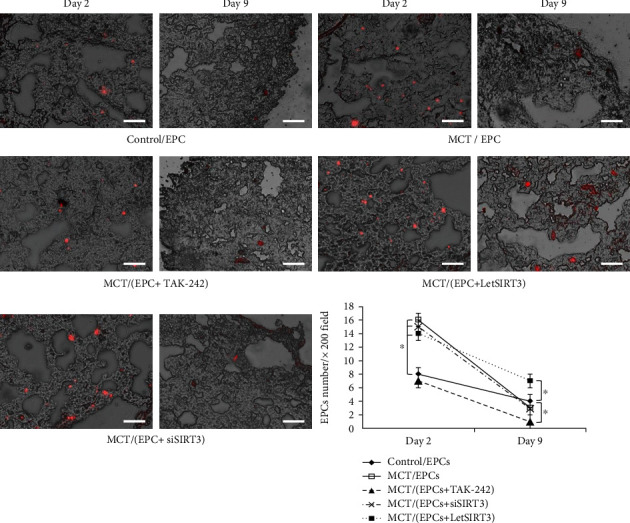
Recruitment and implantation of EPCs in lung tissue with MCT-induced pulmonary arteriolar injury. Samples were harvested 2 or 9 days after EPCs delivery through right jugular vein. EPCs were identified by CMTMR fluorescence (red). Fluorescent and light micrographs were merged and analyzed. Representative photos from day 2 to day 9 of each group and the quantitative comparison of the EPCs per ×200 field among groups (*n* = 4 per group) are shown (Scale bars 100 *μ*m). Data are presented as mean ± SD. ∗*p* < 0.05.

In order to observe the EPCs incorporation and survival in lung tissues after delivery through the right jugular vein, we labeled the EPCs with CMTMR (red fluorescence) which can exist for more than 2 weeks before quenching in vivo [21]. As shown in Figure 7, recruited EPCs can be found significantly increased on day 2 in every group which received the MCT, except TAK-2 pretreated EPCs group, indicating the MCT-induced pulmonary arteriolar injury promoted the recruitment and planting of EPCs through TLR4 at least partly.

However, on day 9, the residency of EPCs in lung tissue was low in almost every group, implying the challenge of relatively long-term existence of transplanted EPCs in inflammatory microenvironment in vivo, whereas we observe a significantly improved EPCs retention in LetSIRT3 EPCs group, which suggested the improved cellular survival rate (Figure 7).

**Figure 8:**
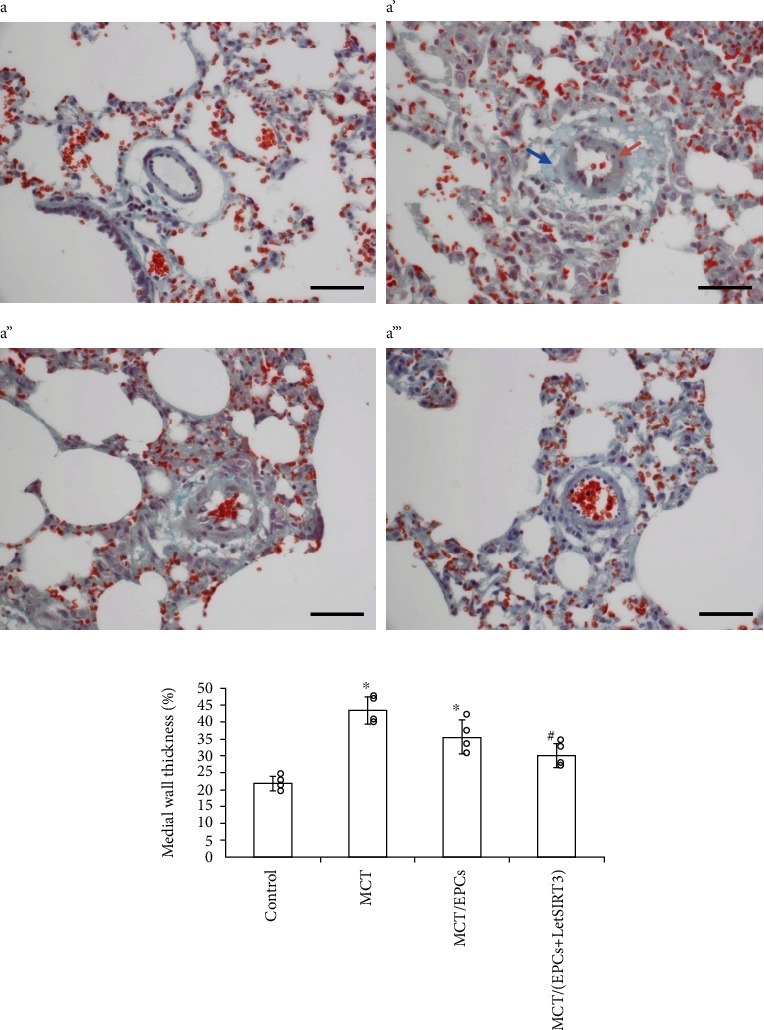
SIRT3 ameliorates the pulmonary arteriolar remodeling after MCT-induced pulmonary arteriolar injury. Three-micrometer lung sections were stained by Masson's Trichrome Stain, by which smooth muscle is indicated as pink (pink arrow), fibril tissues as green (green arrow), cellular nucleus as brown and erythrocytes as red. Representative histological photomicrographs of interacinar pulmonary arterioles (≤100 *μ*m) from each group are shown and analyzed. (a) The lumen of the pulmonary intra-acinar arterioles of the control group was surrounded by thin vessel wall without obvious medium and adventicium. (a') A muscularized interalveolar arteriole from the MCT group with smooth muscle cell circumference (pink arrow) and adventitial hypertrophy (blue arrow). (a”) A partially muscularized arteriole from MCT and EPCs treated group. (a”') An arteriole from the Let-SIRT3(+) EPCs treated group possessing a minor muscular and fibrotic component in the vessel wall (scale bars 100 *μ*m). (b) Quantitative analysis of pulmonary arteriole medial wall thickness 6 weeks after MCT or saline injection. Data are mean ± SD. ∗*p* < 0.05*vs.* control, ^#^*p* < 0.05*vs*. MCT group.

Furthermore, the pulmonary arteriolar remodeling was confirmed by histological analysis on the lung tissues from MCT-treated rats. As shown in Figure 8, the interacinar pulmonary arterioles (external diameters ≤100 *μ*m) of MCT-treated group exhibited a substantially medial hypertrophy after 6 weeks with increased medial wall thickness (21.75 ± 2.2 vs. 43.4 ± 4.1%, *p* < 0.05; Figure 8(b)). EPCs transplantation led to a trend of decreased average medial wall thickness percentage. However, only the LetSIRT3(+) EPC group observed a significant mitigation on medial thickening and adventitial fibrosis, which may result from suppressed inflammation and boosted tissue repair. In respect of the number of arterioles scored by 100 alveoli, EPCs groups failed to raise the density significantly.

## 4. Discussion

In the past decades, EPC has been believed to be an important cell source to promote angiogenesis and tissue regeneration at the site of injury upon the onset of diverse pathologies including ischemic cardiovascular disease, bacterial sepsis, and metabolic disorder [1, 2, 22–25]. However, donor EPCs *in vivo* are usually susceptible to the hostile microenvironment of oxidative stress and inflammation, which leads to low survival rate and dysfunction of the transplanted cells and constitute the major challenges to EPCs therapy [22, 23]. As cell pretreatment or preconditioning has been demonstrated to benefit stem cell transplantation, it has attracted more and more attention in cell-based therapy research [26].

Recently, some authors have reported the expression of functional TLR4, coreceptor CD14, and myeloid differentiation factor (MyD) 88 in human EPCs [12–14]. Activating TLR4 by appropriate dose of LPS, one of the key modulators that mediates cell bioactivity by engaging membrane-bound TLR4 signaling pathways [27], can promote EPCs viability [14], stimulate EPCs adhesion [28], initiate inflammation-induced angiogenesis [29], increase EPCs exosome secretion and paracrine activity [30], and promote EPCs migration in response to chemoattractants such as SDF-1*α* [14]. Our previous experiments revealed that pretreatment with 1 *μ*g/mL LPS for 12 h protected human EPCs from oxidative stress-induced apoptosis and reduced ROS generation [15], whereas LPS at a dose of 10 *μ*g/mL decreased the viability, migration, adhesion, and in vitro angiogenesis of EPCs [13]. All these findings suggest the critical potential of TLR4 signaling pathway in regulating EPCs biological capacity and therefore influencing the efficiency of EPC-based therapy. However, hitherto the evidence of scientific research and the understanding of the underlying molecular mechanism remain inadequate.

Oxidative stress is among the major mechanisms involved in inflammation and ischemic injury. ROS, a range of oxygen-containing species, has been well recognized as a mediator that exerts severe intracellular oxidative stress and prompts an inflammatory response, structural reorganization, and even cell phenotype transition [31]. The functional state of mitochondria plays an important part in cell oxidative damage. On one hand, the mitochondrial redox balance is critical for ROS generation and elimination, and its defects contribute to the abnormal membrane potential and induce the release of proapoptotic protein [32, 33]. On another hand, ROS plays a key role in mediating the mitochondrial membrane permeability and the proapoptotic protein Bax mitochondrial location [34, 35]. In recent years, the potential interesting links between TLR4 signaling and mitochondrial homeostasis have been suggested by several authors' work. Winter et al. reported that HSP 27-mediated TLR4 signaling activation induced adenine nucleotide translocase (ANT) 1 expression and stabilized the mitochondrial membrane potential in cardiomyocytes, which may represent a new mechanism by which ANT1 is part of the cardioprotective HSP27- TLR4 signaling [36]. In another study, Wu et al. reported that TLR4 activation can downregulate the optic atrophy (OPA) 1 in the inner mitochondrial membrane by elevated TNF-*α* level and ROS stress, induce mitochondrial dynamic imbalance and damage, and contribute to the progress of experimental autoimmune myocarditis to dilated cardiomyopathy [37]. However, effects of TLR4 signaling on EPCs mitochondrial function have never been reported before. In the present study, we found that high-dose LPS (10 *μ*g/mL) increased mitochondrial membrane permeability and superoxide generation significantly through the TLR4-dependent pathway in human EPCs at least partly. This can help us to understand the connection between LPS and EPCs oxidation injuries which reported before.

Sirtuin (SIRT) family members were located in nuclear and mitochondrial organelles, maintaining redox homeostasis via regulating the oxidative stress-associated genes [38]. Previous publications revealed that SIRTs might also serve as upstream molecules to regulate the expression of ROS generation genes including NADPH oxidases (Nox) and ROS elimination genes including Mnsod in cells [38, 39]. As mainly distributed in mitochondria, SIRT3 is linked to a variety of oxidative stress-related diseases. The damage of mitochondrial antioxidant network occurs in many oxidative stress-related diseases, which may be reversed by robust SIRT3 function [39, 40]. Considering the important role and unclear mechanism of oxidative stress and mitochondrial dysfunction in EPCs survival, it is interesting to evaluate the involvement of SIRT3 in this process.

We firstly studied the expression of SIRT3 in human EPCs, via the TLR4 pathway, high-dose LPS downregulated the expression of SIRT3, while protective dose LPS upregulated its expression. Further in vitro experiment showed that the SIRT3 deacetylase activity level was positively correlated with the protein expression level. On this basis, we tried to test mitochondrial SIRT3 function by gene modulation. SIRT3 lentivirus was used to induce SIRT3 overexpression in human EPCs, which provided the protection on EPCs from mitochondrial oxidative stress and dysfunction induced by high-dose LPS. It also improves the viability and proliferation of EPC in vitro assays. When considering the reversed expression of SIRT3 after specific blockade of TLR4 [41], we consider that SIRT3 mediates the influence of TLR4 signaling pathway on ROS level and mitochondrial function and contributes to the EPCs survive.

As a key mediator in the inflammation and immune response in tissue injury, TLR4 signaling seems play a crucial and complicated role in vascular homeostasis and remodeling [42]. For example, some studies reported that TLR4 deletion could prevent mice from hypoxia-induced pulmonary hypertension (PH) and vascular wall thickening [43, 44]. Meanwhile, some authors found no effect of the TLR4 deletion from myeloid lineage cells on the development of PH and are concerned about the global deletion of TLR4 which may disturb the vascular homeostasis and bring complicated and uncertain influence to the circulation [10]. One possible scenario is systemic TLR4 activation that will induce severe inflammation and injury to vascular cells and tissue, but appropriate and controllable TLR4 signaling modulation may stimulate the EPC activities and contribute to the homeostasis restoration in vivo.

In the animal experiment, we employed monocrotaline to induce vascular inflammation and subsequent remodeling (medial hypertrophy and adventitial fibrosis) to arterioles in the lung, which has been established and recognized well as previous studies [21]. We observed fluorescence-marked EPCs recruitment in lung tissue on days 2 and 9 after cell injection. Compared to normal lung, planted EPCs increased significantly in MCT- induced lung injury on day 2. Specific TLR4 antagonist TAK-242 significantly reduced this aggregation, which highlighted a close relationship between the activity of TLR-4 and EPCs migration. This is in line with the results of previous in vitro experiments, where the suppression of TLR-4 could abrogate the cell migration in response to activation of the CXCR-4/SDF-1*α* axis [45].

On day 9, we found that EPCs retention in lung tissue only increased significantly in the group treated by let-SIRT3(+) EPCs, suggesting the improved survival and residence of these transplanted EPCs. However, there was no significant increase in EPCs retention in other lung injury groups, including LPS or TAK-242-pretreated EPCs. This suggests the value and necessity to supplement exogenous robust EPCs in the case of vascular injury, because even though the number of circulating EPCs can be increase in response to inflammation [46], the long-term existence of endogenous EPCs in tissue may be hampered by the oxidative stress–induced suppression on cellular activities.

After 42 days development, the MCT induced evident medial hypertrophy and adventitial fibrosis. By Masson staining, the low-dose LPS group showed a relieving trend; however, only the let-SIRT3(+) EPCs group presented a significant protective benefit. This phenomenon could be explained by the enhanced survival of implanted human EPCs in let-SIRT3(+) group, which could provide the spatiotemporal condition for further EPCs-derived benefit by mechanisms including paracrine activity and angiogenesis.

## 5. Conclusion

The data from the current study has provided the evidence for the first time for the critical role of SIRT3 in the LPS-TLR4-induced oxidative stress and mitochondrial dysfunction in human EPCs, which has influence on the cellular viability and proliferation. By in vivo experiments, we showed that TLR4 mediates the early-stage recruitment of transplanted human EPCs in response to pulmonary arteriolar inflammation injury; however, SIRT3 has more important impact on the survival of incorporated EPCs in tissue and thereby contributes more to the vasoprotective antiremodeling effect of human EPCs. The TLR4-SIRT3 signaling in EPCs may represent a new manipulating target for developing efficient cell therapy strategy.

### 5.1. Limitation

The present study also has a few limitations. Firstly, recent studies have demonstrated that LPS can exert its effects though TLR4-dependent and TLR4-independent pathways [47]. Thus, whether the TLR4-independent pathway is also engaged in mitochondrial dysfunction and SIRT3 regulation requires further study. Secondly, the mechanism underlying how TLR4 activation affected SIRT3 expression still needs to be determined. Thirdly, the complicated biological behavior after recruitment and in vivo endpoints of transplanted EPCs need further observation and illustration.

## Data Availability

The data used to support the findings of this study are available from the corresponding author upon request.

## Abbreviations

ANT: Adenine nucleotide translocase
CCK-8: Cell Counting Kit-8
CMTMR: 5-(and-6)-(((4-chloromethyl)benzoyl)amino) tetramethylrhodamine
DiI-Ac-LDL: 1,1-dioctadecyl-3,3,3,3-tetramethylindocarbocyanine-labeled acetylated low-density lipoprotein
EPC: Endothelial progenitor cell
FITC-UEA-1: Fluorescein isothiocyanate-labeled Ulex europaeus agglutinin lectin-1
H_2_DCFDA: 2′,7′-dichlorodihydrofluorescein diacetate
HRP: Horseradish peroxidase
HSP: Heat-shock proteins
LetSIRT3: Recombined lentivirus of SIRT3
LPS: Lipopolysaccharide
MCT: Monocrotaline
mPTP: Mitochondrial permeability transition pore
MyD: Myeloid differentiation factor
Nox: NADPH oxidases
PBMNC: Peripheral blood mononuclear cell
PVDF: Polyvinylidene fluoride
SIRT: Sirtuin
siSIRT3: siRNA of SIRT3
TLR: Toll-like receptor.

## Supplementary Materials

Supplementary Materials Supplementary Materials 1 Identification of human EPCs. (a) The human EPCs showed the typical morphological transformation from adherent round PBMNCs on day 2 of culture, to spindle shape on day 4, and then to cobblestone-whorl-like arrangement after 10 days. (b) Green color showed FITC-UEA-I-positive cells (exciting wavelength 477 nm) on day 10. Red color represented Dil-ac-LDL-positive cells (exciting wavelength 543 nm). Yellow-colored double-positive cells in the overlay were identified as differentiating EPCs (×400) (Scale bars 100 *μ*m).
