# Supplementary figures and images for: TLR4-SIRT3 Mechanism Modulates Mitochondrial and Redox Homeostasis and Promotes EPCs Recruitment and Survival
Source: Oxid Med Cell Longev. 2022 Jul 4;2022:1282362. doi: 10.1155/2022/1282362 (PMC9273456; doi:10.1155/2022/1282362)

**Supplementary**

**a**


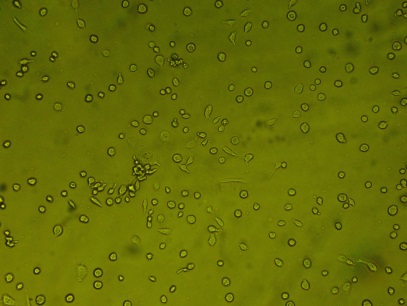

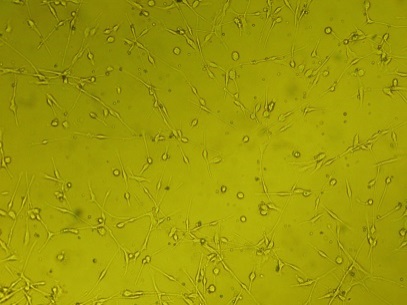

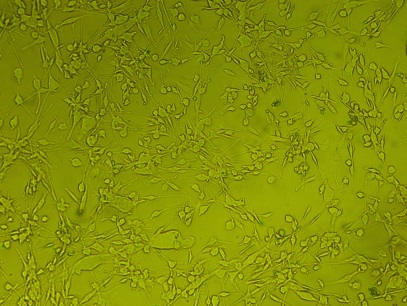


**Day 10**

**Day 4**

**Day 2**

**b**






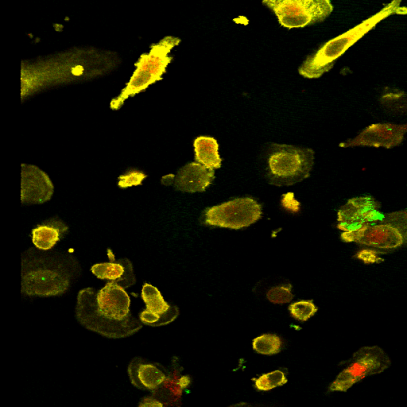

Supplement: Supplementary Materials — 1 Identification of human EPCs. (a) The human EPCs showed the typical morphological transformation from adherent round PBMNCs on day 2 of culture, to spindle shape on day 4, and then to cobblestone-whorl-like arrangement after 10 days. (b) Green color showed FITC-UEA-I-positive cells (exciting wavelength 477 nm) on day 10. Red color represented Dil-ac-LDL-positive cells (exciting wavelength 543 nm). Yellow-colored double-positive cells in the overlay were identified as differentiating EPCs (×400) (Scale bars 100 μm). [file 1282362.f1.docx]
